# Assessing Training Needs and Self-Efficacy of Hospital Administrators in Western India

**DOI:** 10.7759/cureus.75292

**Published:** 2024-12-07

**Authors:** Jallavi Panchamia, Litty Denis, Namitha Ashwath

**Affiliations:** 1 Department of Health Policy, Management and Behavioral Science, Indian Institute of Public Health Gandhinagar, Gandhinagar, IND; 2 Department of Social Sciences, Central University of Gujarat, Gandhinagar, IND

**Keywords:** competencies, hospital, hospital administrators, managers, self-efficacy, training needs assessment

## Abstract

Introduction: Effective training is required to enhance hospital administrators' abilities and improve performance. This study evaluates the training needs and their association with the self-efficacy of hospital administrators in public and private hospitals in Western India.

Methodology: This study evaluated the training needs of hospital administrators using a survey-based methodology and a descriptive cross-sectional design. The study included hospital administrators from public and private healthcare institutions in Western India. Purposive selection strategies were used to choose 127 administrators/managers to participate in the study. Primary data was gathered using a validated measure of general self-efficacy scale by Schwarzer and Jerusalem and the WHO-adopted Hennery-Hicks Training Needs Assessment Questionnaire with 30 items. These items were categorized into six core categories: communication, self-management, hospital operations, research and development, information and communication technology (ICT) and report management, and team management and supervision. Statistical analysis included descriptive statistics, Pearson’s correlation to evaluate the association between training needs and self-efficacy, and a t-test to compare the administrators of the private and public sectors.

Results: The findings indicated strong positive associations between training needs and self-efficacy in administrators. There was higher self-efficacy among private sector administrators (M: 4.08, SD: 0.50) than their counterparts in the public sector (M: 3.91, SD: 0.60). Key areas for the training requirement included self-management skills (93.7%), ICT and report management skills (92.0%), and team management skills (93.2%). Additionally, managers in the private sector were more likely than those in the public sector to say they needed more training.

Conclusion: The findings showed a considerable demand for self-management and ICT and report management skills training. Also, hospital administrators showed a significant willingness to participate in training to advance their abilities despite their degree of experience, indicating a proactive commitment to professional development.

## Introduction

Employee training often becomes a backburner in the busy management of healthcare services due to numerous competing issues in hospital settings. However, developing and retaining the best human resources and providing essential new skills and knowledge is crucial. This effort aims to develop a skilled workforce of mid-level administrators who are integral to improving the effectiveness and efficiency of administrative tasks in hospitals. Their contributions are vital to ensuring the delivery of high-quality healthcare services [1].

Physicians also serving administrative roles are well-versed in the legal framework governing patient care and clinical duties [2]. With a concentration on management rather than direct patient care, these professionals manage the day-to-day operations of healthcare facilities. They are typically armed with bachelor's or master's degrees in hospital administration. Depending on the business, a healthcare administrator's responsibilities may vary, including environments like physical therapy clinics, nursing homes, and operating rooms. Notwithstanding these differences, a few fundamental duties are always the same, which include establishing and overseeing medical staff and workforce duty schedules, taking care of the facility's budget and financial issues, overseeing the billing processes for patients and controlling expenses, maintaining and improving the standard of the services provided by the facility while cultivating an environment that is restorative and ensuring adherence to all applicable laws and regulations. Other essential duties include presenting operational performance, making financial reports, strategic planning, investing in investor meetings, interacting with governing boards to support strategic decision-making, monitoring expenditure trends, and following financial restrictions [3].

The need for improved effectiveness and productivity has become pivotal for hospital-quality service outcomes [3]. Practical training requires an hour, and the role played by staff training and development can no longer be overlooked [4,5]. Usually, before training programs are organized, efforts should be made through individual and organizational appraisals to identify the training needs. The training needs assessment is critical for the training and development function. Very few studies have reported the results of training needs assessments of hospital administrators representing private and public healthcare organizations in Western India [6]. The present study attempts to address the realized gap to identify the managerial training needs of hospital administrators working in different hospitals in Western India. Further, the study aims to explore three key research questions: (a) What are the perceptions of hospital administrators regarding the competencies that should be the focus of training programs? (b) What are their views on the effectiveness of training programs in enhancing their roles? (c) How does the belief in the necessity of training for a specific competency influence their perceived self-efficacy?

Literature review

In an ever-evolving and high-pressure healthcare environment, hospital administrators’ roles have grown increasingly high-stakes, necessitating resilience, specialized skills, and a strong sense of self-efficacy. Self-efficacy is one’s belief in accomplishing tasks and overcoming challenges [7]. It has been shown to impact overall job performance and motivation significantly. This belief influences the efforts put forward and their level of achievement [8-11]. In healthcare settings, self-efficacy bolsters an individual’s capacity to perform and protects against stress and burnout. A study on healthcare, where administrators must often make quick, high-impact decisions, shows that self-efficacy influences the performance of individuals at the workplace; higher levels of self-efficacy lead to enhanced motivation and improved performance [12].

During the COVID-19 pandemic, self-efficacy was crucial in healthcare, where administrators faced unprecedented challenges in crisis response, communication and coordination, and resource management. A rapid review of healthcare practitioners during the COVID-19 pandemic revealed strong relations between resilience and self-efficacy, helping them cope better with crisis demands [13]. This mediated a sustained team’s performance withstanding, adapting to the challenging circumstances, and contributing to the organization’s resilience, resulting in better patient care.

Besides crisis management during emergent situations, self-efficacy plays a significant role in job satisfaction and employee retention within healthcare organizations. Self-efficacy was reported to be higher among hospital administrators with a strong commitment to their job roles, greater stress resistance, and a sense of job satisfaction [13,1]. Also, research conducted at Kerman University on the administrative staff revealed that self-efficacy and emotional intelligence enhance positive workplace relationships and job satisfaction. These findings imply the need for training, counseling, and retention of administrative personnel to improve the medical facility's performance [8]. It is essential to manage the interpersonal dynamics of hospital administrators, especially in complex healthcare environments where teamwork and collaboration are crucial.

Previously published literature shows paramedical and non-medical department staff members perceive managerial training needs, emphasizing leadership, interpersonal interactions, and various patient, supervisor, and management-related aspects [14,15]. These investigations show that respondents from both strata have difficulties relating to management and superior variables; personality-related and interpersonal interaction problems notably impact respondents from non-medical departments. Rajan's study on the training requirements for health professionals revealed a number of important gaps in the domains of behavior and function. Among other behavior-related issues, they indicated a critical need for training in handling criticism, stress management, and office politics [15]. Critical training needs also included function-related characteristics such as multitasking under pressure, making decisions without senior direction, and utilizing resources.

Parallel to these findings, Gaspard and Yang's work in the setting of developing nations emphasized the necessity for training the administrators’ skills in computer literacy, clinical skills, management, effective communication, and research methodologies. Respondents preferred practical training interventions to improve interdepartmental performance [4]. They also emphasized the importance of ongoing education and training needs assessment [16]. Significant training gaps were found in the nursing staff and district medical officers surveyed by Devi and Rao [5]. These gaps were especially evident in teamwork and excitement for learning. This offers important insights into skill inadequacies across multiple domains within healthcare organizations, even though planning, organizing, and communication abilities showed somewhat reduced training demand gaps.

Furthermore, research has found positive outcomes in the efficacy of training programs. The communication skills training course by Nørgaard et al. showed that healthcare workers' self-efficacy significantly increased after the training and remained elevated for six months [17]. Similarly, a systematic review by Mata et al. demonstrated how training programs, especially those emphasizing conceptual concerns and practical learning, can effectively improve healthcare workers' self-efficacy, attitude, and behavior. When taken as a whole, these results highlight the complexity of evaluating training needs in the healthcare industry and the potential benefits of focused training interventions in raising performance and professional capabilities [18].

The existing literature highlights the critical influence of self-efficacy and targeted training programs on the impactfulness of hospital administrators. The review emphasizes the value of developing self-efficacy and providing adequate training for managers to build their resilience and strong teams to meet the demands of patient care and organization. Most of the studies conducted are not in the Indian context, where hospital administrators face multifaceted environments, and hardly any studies have focused on the need assessments of hospital administrators in government and private hospitals in India. The current study aims to expand the existing literature and provide insights to inform future training efforts and recruitment decisions to ensure competent hospital administration in public and private settings.

## Materials and methods

The study focuses on the training needs of the administrators/managers in both public and private hospitals. This survey-based study adopted a descriptive cross-sectional design frequently used to investigate patterns and interactions within a population at a particular moment in time [19]. A purposive sampling technique was used to choose research participants, considering the designated hospital administrators and non-designated personnel in charge of hospital administration, i.e., those who own the roles and responsibilities of the hospital administrator. Based on inclusion criteria, 145 hospital administrators/managers working in hospitals in Western India were included in the study. Administrators from both the public and private health sectors were a part of it. Participants who did not fully complete the survey and other healthcare professionals not in managerial roles were excluded. The collected data was screened for completeness, and eventually, 127 responses were considered for further analysis upon administrators removing 19 incomplete responses, resulting in an approximate 87.5% response rate.

After obtaining the necessary permissions and receiving ethical approval from the Ethics Committee of the Indian Institute of Public Health (approval number: 2020-21/031/42), all respondents were informed of the study's objectives, and consent was obtained electronically. The primary data was collected through an online survey. The survey instrument used in this study was a structured questionnaire divided into three sections. The first part was to understand the training needs through an assessment tool developed by the WHO [16]. This tool assessed the participants’ attitude toward training. It has 30 questions structured across six themes: communication skills, self-management skills, team management and supervision skills, research and development skills, information and communication technology (ICT) and report management skills, and hospital operations skills. The questionnaire is extensively utilized as a validated instrument by the WHO, which helps identify customized needs for training to address professional growth [16]. The second part of the questionnaire focused on the self-efficacy scale by Schwarzer and Jerusalem [20]. Self-efficacy was measured using 12 statements, including a sample question: “I can usually handle whatever comes my way.” Section 3, titled Demographic Profile, included questions about the respondent's demographic information, such as type of organization, designation, and work experience (Table 1).

**Table 1:** Demographic profile of the respondents

Measure	Description	Frequency	Percentage
Age	21-30 years	69	53.9
	31-40 years	48	37.5
	41-50 years	9	7.0
	More than 50 years	1	0.8
Gender	Male	72	56.7
	Female	55	43.3
Qualification	Masters in healthcare management and allied courses	111	87.4
	Other post-graduate degree	2	1.6
	Multiple post-graduate degree	4	3.1
	PhD	1	0.8
	Graduate	9	7.1
Work experience	Below 2 years	61	48.0
	Between 3-5 years	25	19.7
	Between 5-10 years	21	16.5
	More than 10 years	20	15.7
Type of organization	Public hospital	47	38.0
	Private hospital	80	63.0

Data from 127 participants was analyzed through descriptive statistics to summarize the demographic characteristics of the respondents and the training needs identified. The study utilized Pearson’s correlation coefficient to explore the relationship between self-efficacy and training needs. Also, an independent t-test was conducted to assess differences in self-efficacy levels between public and private hospital administrators. The reliability of the responses was checked using Cronbach’s alpha.

## Results

Table 1 shows that 56.3% of the respondents were male of all the participants, and the majority were from the age group between 21 and 30 years, indicating that a significantly young portion of hospital administrators and overwhelmingly 87.4% of participants had a master’s degree in healthcare management. Nearly half of the participants (48.0%) had less than two years of work experience, indicating the predominance of early career professionals.

Training needs and competency gaps in hospital administrators

**Figure 1:**
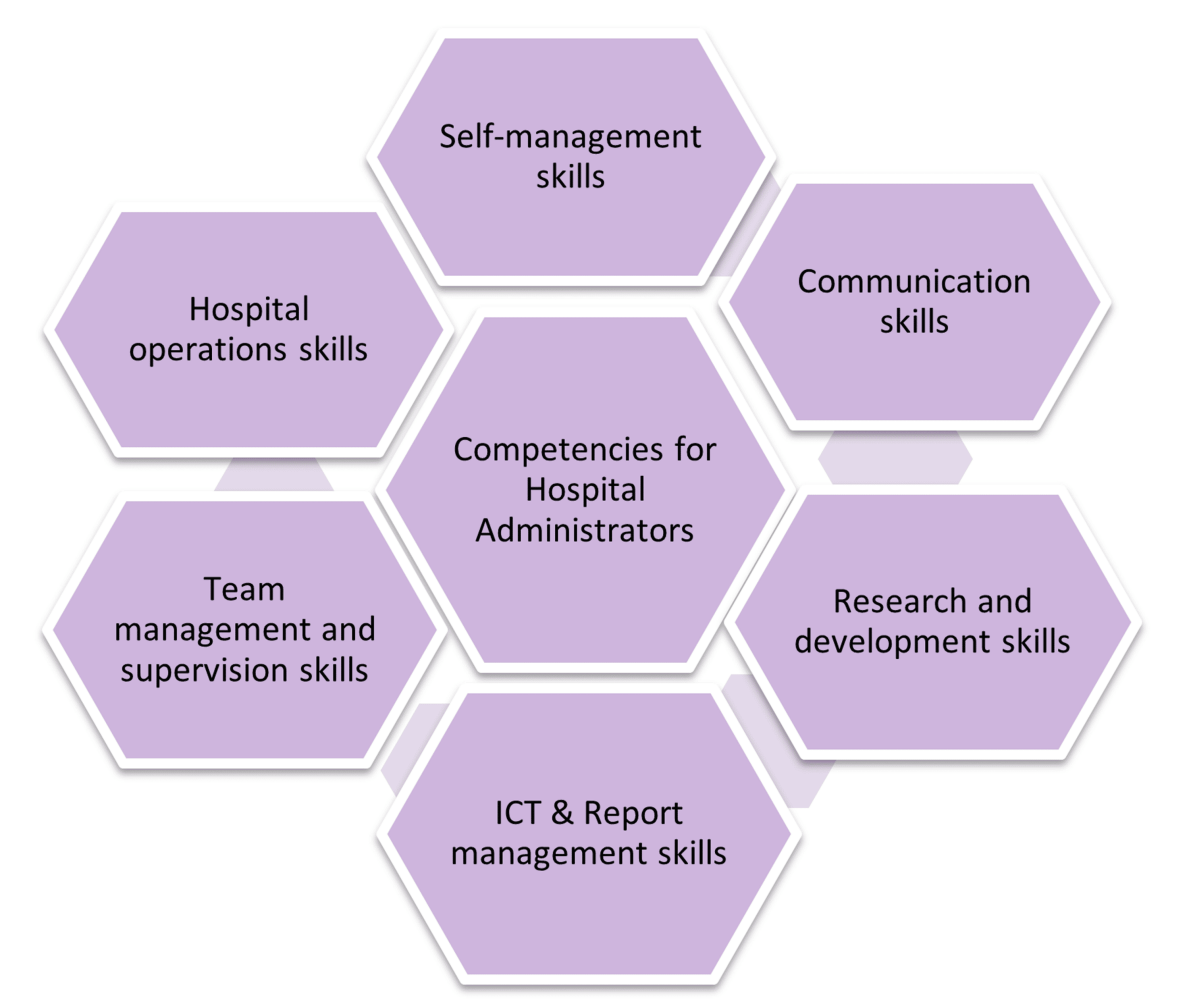
Competencies for hospital administrators ICT: Information and communication technology Hennessy and Hicks (2011) [16]

The competencies that hospital administrators must possess are diverse tasks and obstacles they face daily. Results indicated a greater mean score in self-management competencies, including emotional intelligence, work-life balance, and organizing skills [20]. Other areas, too, reported higher needs, as shown in Table 2. It also indicated that private managers reported higher perceived training needs in all six domains of management training than government healthcare organization administrators. Figure 1 indicates all the domains of the training needs for hospital administrators adapted from Hennessy and Hicks [16].

**Table 2:** Reliability test for competency variables ICT: information and communication technology

Hospital management competency variables	No. of items	Cronbach's alpha	Mean score (private sector)	Mean score (public sector)	% of respondents who gave a rating above 3
Communication skills	4	0.784	3.98	3.70	86.6
Self-management skills	4	0.71	4.23	4.11	93.7
Hospital operations skills	6	0.874	4.12	4.07	89.5
Team management and supervision skills	6	0.798	4.11	4.10	93.2
Research and development skills	8	0.82	3.56	3.50	91.2
ICT and report management skills	4	0.668	4.08	4.06	92.0
Self-efficacy	12	0.776	4.08	3.90	94.5

Data was tested for reliability using Cronbach's alpha test. Table 2 indicates the mean score and reliability statistic of competency variables. The results indicate that hospital administrators have a substantial need for training in these six important areas. Six categories represent hospital administrators' competencies, which indicate the diverse daily tasks and obstacles they face.

Communication Skills

According to 86.6% of administrators, training is necessary to communicate effectively with patients, caregivers, co-workers, and superiors, as they gave a rating of more than 3 in the communication domain of the training needs assessment tool. This skill was perceived as an essential area for training. Hospital administrators should communicate proficiently with patients, visitors, superiors, and other staff members. The difficulty often arises when the hospital administrators, patients, or attendants cannot understand one another. The hospital administrators should be proficient in the language and have communication abilities.

Self-management Skills

There is a strong need for instruction on accepting accountability for personal and professional life. This need was among the highest felt needs by 93.7% of the participants. Self-management abilities enable people to effectively control and regulate their emotions, thoughts, and behavior in various contexts. Strong self-management abilities enable workers to independently define goals and make every effort to meet them on time. Time management, appraising one’s own performance, and personally coping with the changes are some of the indicators that define the self-management skills of an employee.

Hospital Operations Skills

According to administrators, the reported need was higher (89.5%). Such training is crucial for supervising regular hospital procedures, managing resources, and determining the needs of patients. Monitoring and managing all practices set up to cover areas like facilities, staffing, policy, and financing to drive the various processes that result in the services delivered. These processes include quality control, care coordination, staff certification and licensing, credentialing, risk management for health insurance and associated claims, medical review organizations, legal, auditing, and compliance programs.

Team Management and Supervision Skills

There is a high demand (93.2%) for training in information sharing, task delegation, dispute resolution, and successful team players. "Team management" describes the strategies, tactics, and procedures used to lead, direct, and coordinate a group of people to carry out a particular activity. Goal setting, performance management, communication management, and other aspects of teamwork are all included in team management. Managing disagreements within the team also includes handling interpersonal issues.

Research and Development Skills

Respondents felt a strong demand (91.2%) for instruction in carrying out research tasks, gathering and analyzing data, and applying research results to practical situations. Research and development refers to organizations' processes to create and launch new products or services. Interpreting one’s research/study findings, applying research results to your practice, accessing relevant literature for your clinical/managerial work, statistically analyzing your data, undertaking health promotion studies, collecting and collating relevant information, designing a research study, data collection, and analysis are a few examples of research and development skills.

ICT and Report Management Skills

Training in ICT and report management skills was necessary for 92.0% of participants, which included using technical equipment, HMIS administration, and report writing. The capacity to communicate with people using various technologies is referred to as ICT abilities. ICT, like information technology, refers to using technology for routine, everyday activities, including sending emails, making video calls, conducting internet searches, utilizing tablets or mobile phones, and more. ICT proficiency may also involve the capacity to use more traditional communication mediums like telephones, radios, and televisions.

Self-efficacy Among Hospital Administrators

As mentioned in Table 2, 94.5% of hospital administrators perceived that they have a high self-efficacy. This belief can be strengthened with practical training and a favorable attitude toward the training programs, which can help one gain more confidence in dealing with difficult situations. Irrespective of the organization set up, government or private, both respondents did not correlate their perception of self-efficacy with their organization's structure.

Attitude toward the training effectiveness

Interestingly, 92.1% of the respondents had a favorable attitude toward training given in organizations to increase their skill set, whereas 7.9% had an unfavorable attitude toward them. The participants were asked about their perception of online training in comparison to traditional training; 42.5% of the respondents disagreed that online training is more effective than traditional or offline in-person training, 21.3% of the respondents were neutral about it, and 36.2% felt that online training is comparatively more effective than traditional training (Table 3).

**Table 3:** Attitude and perception toward training

Training aspect	Response	Percentage
Attitude toward the effectiveness of organizational training	Favorable	92.1
	Unfavorable	7.9
Perception toward online and traditional training practices	Online training is more effective	36.2
	Neutral	21.3
	Online training is less effective	42.5

Self-efficacy in public and private organizations

An independent sample t-test was conducted to understand the significance of self-efficacy scores between private and public organizations. Levene’s test for equality of variances indicated no significant difference in both groups (F (1,127) = 0.422, p = 0.517), allowing equal variances to be assumed. The results indicated that private hospital administrators reported slightly higher self-efficacy (M: 4.08, SD: 0.50) than public administrators perceived self-efficacy (M: 3.90, SD: 0.60). However, statistical comparisons showed no significant difference in self-efficacy (t (127) = -1.682, p = 0.095) between participants in public and private organizations (Table 4).

**Table 4:** Independent t-test results for self-efficacy by type of organization

Type of organisation	N	Mean	Std. deviation	Std. error mean	p-value
Private hospital	80	4.0851	0.50165	0.07317	0.095
Public hospital	47	3.9094	0.60387	0.06751	0.080

**Table 5:** Correlation between perceived training competency and self-efficacy and work experience Values indicate the Pearson's correlation coefficient, ** p < 0.001 (CI 99.9%), * p < 0.05 (CI 95%) ICT: information and communication technology

	Self-efficacy	p-values	Work experience	p-value
Communication skills	0.460**	0.0006	-0.144	0.115
Self-management skills	0.475**	0.0004	-0.075	0.332
Hospital operations skills	0.470**	0.0005	-0.170	0.072
Team management and supervision skills	0.479**	0.0004	-0.192^*^	0.029
Research and development skills	0.404**	0.0009	-0.166	0.080
ICT and report management skills	0.454**	0.0008	-0.051	0.562
Attitude toward training	0.547**	0.0002	-0.083	0.477

Self-efficacy and its association with perceived training needs

As shown in Table 5, self-efficacy was significantly associated with all the perceived training needs of hospital administrators. Precisely, all six skills: communication (r = 0.460, p = 0.0006), self-management (r = 0.475, p = 0.0004), hospital operations (r = 0.470, p = 0.0005), team management and supervision (r = 0.479, p = 0.0004), research and development (r = 0.404, p = 0.0009), ICT and report management (r = 0.454, p = 0.0008), and attitude toward training (r = 0.547, p = 0.0002) indicate that hospital administrators with higher self-efficacy felt the need for training moderately higher than those with low self-efficacy.

Contrary to these findings, the hospital administrators' work experience level was observed to have no significant relationship with their training needs or attitude toward training, i.e., communication: r = -0.144; self-management: r = -0.075; hospital operations: r = -0.170; and ICT and report management: r = -0.051, with p-values greater than 0.05. However, there was a significant correlation between team management and work experience (r = 0.192, p = 0.029), although it was a negligible negative relationship, which could mean that the higher the level of work experience, the lower the need for training on team management.

## Discussion

Training needs assessment

The study shows the six skills or competencies that illustrate the areas in which hospital administrators perceive the professional needs to work and the areas in which they are most robust: communication, self-management, team management and supervision, research and development, ICT and report management, and hospital operations. These competencies were developed using the Hennessy-Hicks Training Needs Analysis Questionnaire and literature review indicators, where hospital administrators might perceive the training requirement to be self-efficient in their work. As seen in the results, administrators perceived a high need for training in almost all areas; more than 90% of respondents perceived a higher need for self-management, team management and supervision, research and development, and ICT and report management. Similarly, 86% of respondents had higher communication, and 89% had hospital operational skills training needs. Training needs can be as functional as knowing the efficient way of utilizing the resources, handling equipment, dealing with patients, maintaining the hospital's quality standards, difficulty carrying out multiple works simultaneously, and so on, as determined in Rajan’s study [15]. The study found that hospital administrators are like managers who must be able to do it all, so training needs not only in functional areas are high but even higher in behavioral areas, as not all administrators necessarily be good at tolerating criticism of co-workers or supporting other staff, or even feel challenging to cope with office politics, changes in policies, and conveying grievances to higher officers [12].

Hospital administrators' training needs can vary in different settings depending upon their job description, demands and challenges of the position and organization, and their perception and evaluation of themselves. However, no significant difference was seen between the training needs of government and private hospitals, as shown in Table 2. It is as complex as it is to judge how respondents determine their training needs and prefer any training that would help them practically perform in their respective departments. It can be said from the definition and literature that there are defined skill sets hospital administrators must have to be self-efficient in their work [11].

We can only hope to accomplish the millennium development goals by attending to the training requirements of healthcare professionals. Training that meets demands is critical, especially in developing nations with few resources. Every healthcare professional has different and varied needs. Their perceived and actual needs may alter with time, place, and responsibilities. The environment in which professionals operate and the resources available to carry out different activities can affect needs differently. Determining the training needs of administrators requires researchers to perform a needs assessment to decide on the training programs' content [21].

Training programs are designed to fulfill these training needs of hospital administrators, for which the needs assessment is crucial. The program is considered successful when it positively affects the participants or when they adopt these skills. This, in turn, helps them get better and more productive at work. However, a lot depends on hospital administrators' attitude toward these trainings and their effectiveness. To bring out participants’ positive or fairer attitudes toward training, it becomes essential to design the program based on assessment to attract participants' interest and give them an effective experience [22]. In this study, 92% of respondents had a favorable attitude toward training and its effectiveness, which can be interpreted as most participants were inclined toward receiving training or, at the very least, had no problem or negative perception toward the effectiveness of the training, which can later be considered as one of the factors while evaluating the success of the training program. Most participants felt that the training they had attended in the past had been helpful for them in their professional lives and that they could apply it at work as the time they spent on these trainings was worthwhile. No significant difference was seen when analyzing whether there was a difference or commonality in the perception of government hospital administrators and private.

Self-efficacy and its interplay with training needs

Devoting time and energy to learning at work is very important for achieving growth and success in professional life. It determines one’s potential and determination to become self-efficient. It was seen in the study that the higher the training needs, the higher the self-efficacy. This term refers to belief in one's ability to succeed in specific situations or accomplish a task [5]. This can mean that when a manager’s attitude toward training themselves in a specific activity or skill is updated, there is a moderate chance that their self-efficacy is high. This shows their potential and willingness to learn and update their skills. It can be considered one of the significant predictors of their productivity. Higher self-efficacy can also benefit the organization; in this case, the hospital administrator considers himself/herself capable of achieving tasks required to manage the hospital. It was seen in Nørgaard et al.’s study that training can also help increase self-efficacy among healthcare professionals. So, it can be said that the two might be interrelated, as when a participant is self-efficient, he/she can have a positive inclination toward training [17]. When given training, the participant can become more self-efficient. Similarly, in a systematic review study on training programs, trained groups showed improvements in self-efficacy. It was also observed that the programs that approach the conceptual issues and promote the space for experiential learning could be effective in professional communication skills training [18].

We often might assume that with more work experience comes more self-efficiency at work. However, this study showed no significant correlation between self-efficacy and work experience, except for the negative correlation between team management and work experience, as mentioned in Table 5. It can be said that work experience does not affect an individual’s confidence and ability, as one might be on the job for years but still lacks the capability of handling all that comes their way. So, when training needs were observed with work experience, there was no correlation, as it is already stated that irrespective of the years an administrator has worked in the hospital [23], his/her training needs can also be higher. As also seen in the study, administrators with growing work experience may even have higher training needs to keep updated with the new ways a hospital can be managed.

Implications for practice

An important skill that hospital administrators must have is to manage people, as they need to address co-workers of all levels, starting from the chief medical officer or medical superintendent to support staff. One must have team management skills and align the team to manage such a workforce. This study showed a significant correlation (p < 0.05) between team management and work experience. However, it was a negligible negative association, which could mean that the higher the level of work experience, the lower the need for training on team management, i.e., with more work experience, the team management of an individual can get better. So, it was seen that the training needed for team management was low in participants with higher work experience.

Even for an experienced hospital administrator, it can be difficult to manage the daily challenges of managing a hospital, government or private, considering how healthcare demands are increasing daily. When asked about significant challenges faced by having the job of the hospital administrator, the majority of the sample population addressed the communication gap, limited resources, time management, work-life balance, less authority, and inadequate human resources, among others, as some of the significant challenges they face. The respondents commonly reported workload, time management, and patient satisfaction issues.

It was observed that almost all the respondents had undergone training programs related to hospital administration in the past. The most common training to be taken recently was disaster management or hospital preparedness for an emergency by government hospital administrators, whereas National Accreditation Board for Hospitals and Healthcare Providers training was received by private hospital administrators [6]. The most commonly seen training required by the respondents of the government hospitals was finance management computer training as in ICT training and refresher training on government policies and regulations, whereas for the private hospitals, training on stress management and how to balance work and life was observed to be dominant over others. A few other training the respondents required were documentation skills, data analysis, and medico-legal topics. Government and private hospitals' most demanded and common requirement was leadership, human resources, and communication skills training. The study also addresses the hospital administrators' perception of online training in that they are better or more preferable than offline training, to which 42.5% disagreed, and around 21.3% had a neutral response. So, it can be said that more respondents preferred offline training over online training. If we observe them, training needs, attitude toward training and its effectiveness, and self-efficacy correlate. Training needs can be higher if the attitude toward training is favorable; likewise, the greater the need, the higher the self-efficacy, and vice versa.

Strengths, limitations, and further implications

The study has some unique strengths, presenting the area largely unexplored: hospital administrators in Western India. Also, the study compares the private and public healthcare sectors, providing insights into the training needs across these organizational setups. It also explores a crucial yet unexplored area of correlating self-efficacy and training needs vital in designing targeted interventions under healthcare leadership. Although the study presents some unique strengths, it has presented some limitations in hospital administrators’ self-efficacy and training needs that could be overcome with further research. The study was conducted in Western India, so the results cannot be generalized to other parts. Administrative, cultural, and organizational differences influence the results in the broader context.

Further research could expand the geographical scope to include other parts of the country, which could help build a comprehensive understanding of the topic. Another limitation was that the study sample size was limited to 127 participants, and voluntary participation introduced selection bias. This moderately small sample may not be large enough to fully assess diverse hospital administrators' self-efficacy and training needs across Western India.

A large sample size could improve the study's statistical power and yield more robust and generalizable conclusions regarding hospital administrators' self-efficacy and training needs. This could help mitigate the bias on sample size and ensure the reflexivity of the findings in a wider range of contexts for hospital administrators. Focusing solely on hospital administrators' needs neglects the relevance to other healthcare contexts, such as teaching hospitals, nursing homes, and different administrative structures and responsibilities. Hence, future research could bring a more comprehensive understanding of the training needs of the whole health sector.

## Conclusions

The study reveals a high need for training in the six defined competencies among hospital administrators, irrespective of the type of hospital. Effective in-person and offline training programs on upgrading these competencies among hospital administration can be designed and conducted as they have a positive attitude toward training and its effectiveness. However, training needs assessment is essential before designing training programs for a given sample.

Hospital administrators working in various settings are well aware of their challenges and job requirements and are seen to have high self-efficacy as professionals. They are not only aware of their capabilities but also know where they lack and are willing to undergo training to improve their skills despite their level of work experience. They have a more significant role in the hospital's functioning than they are given credit for. So, focusing on their needs is also critical to improving the hospital.
